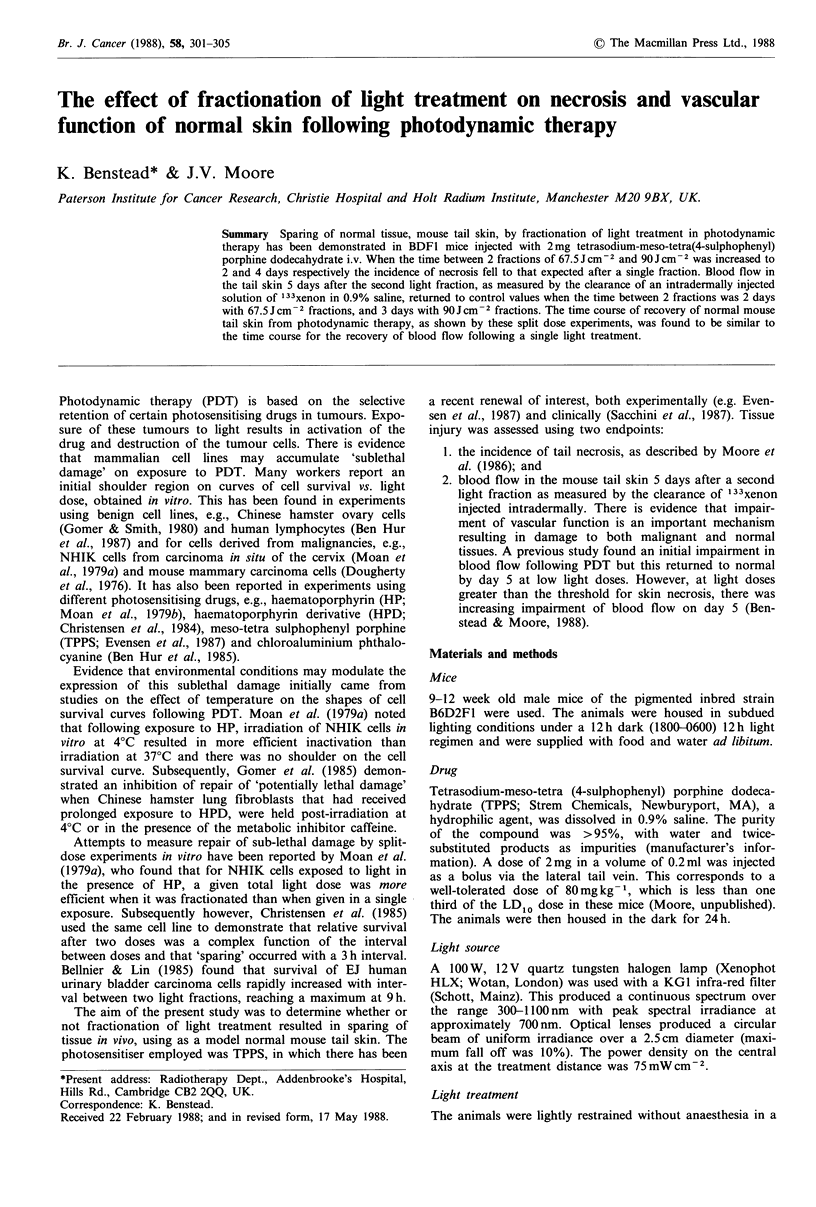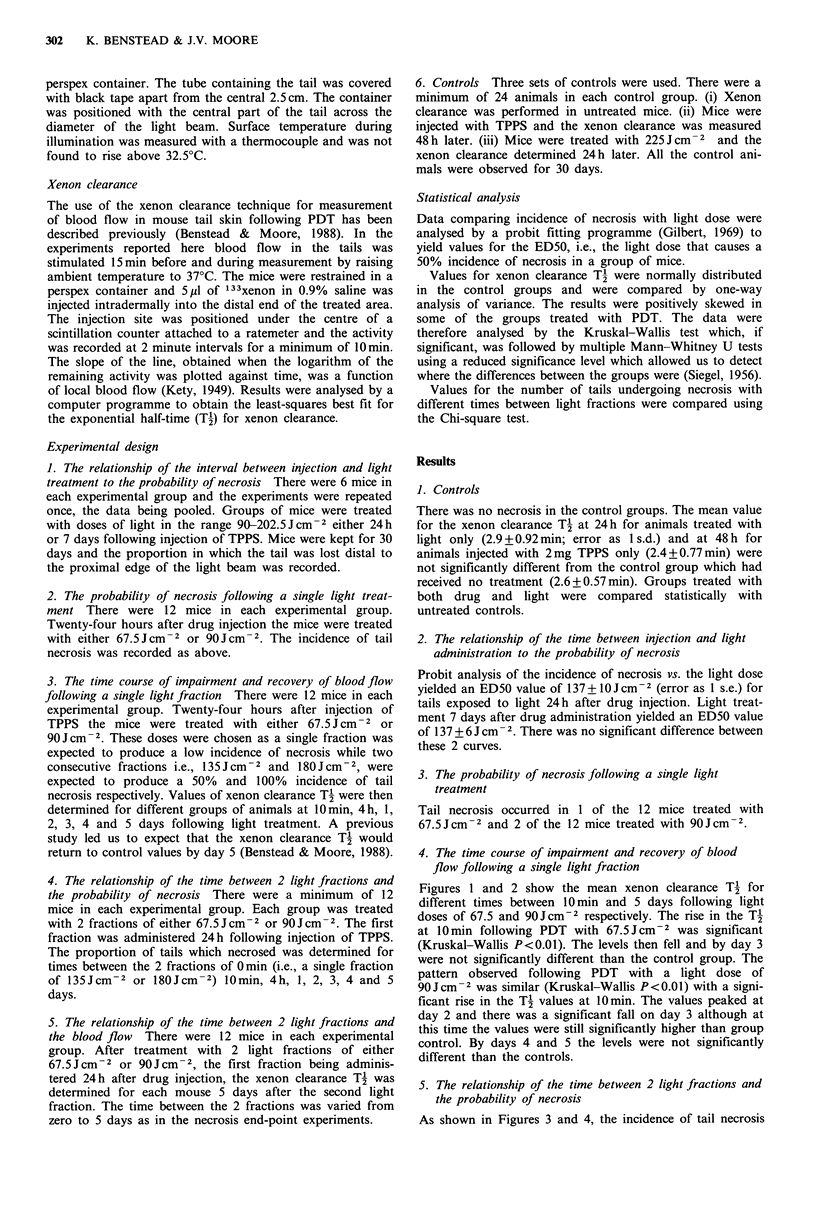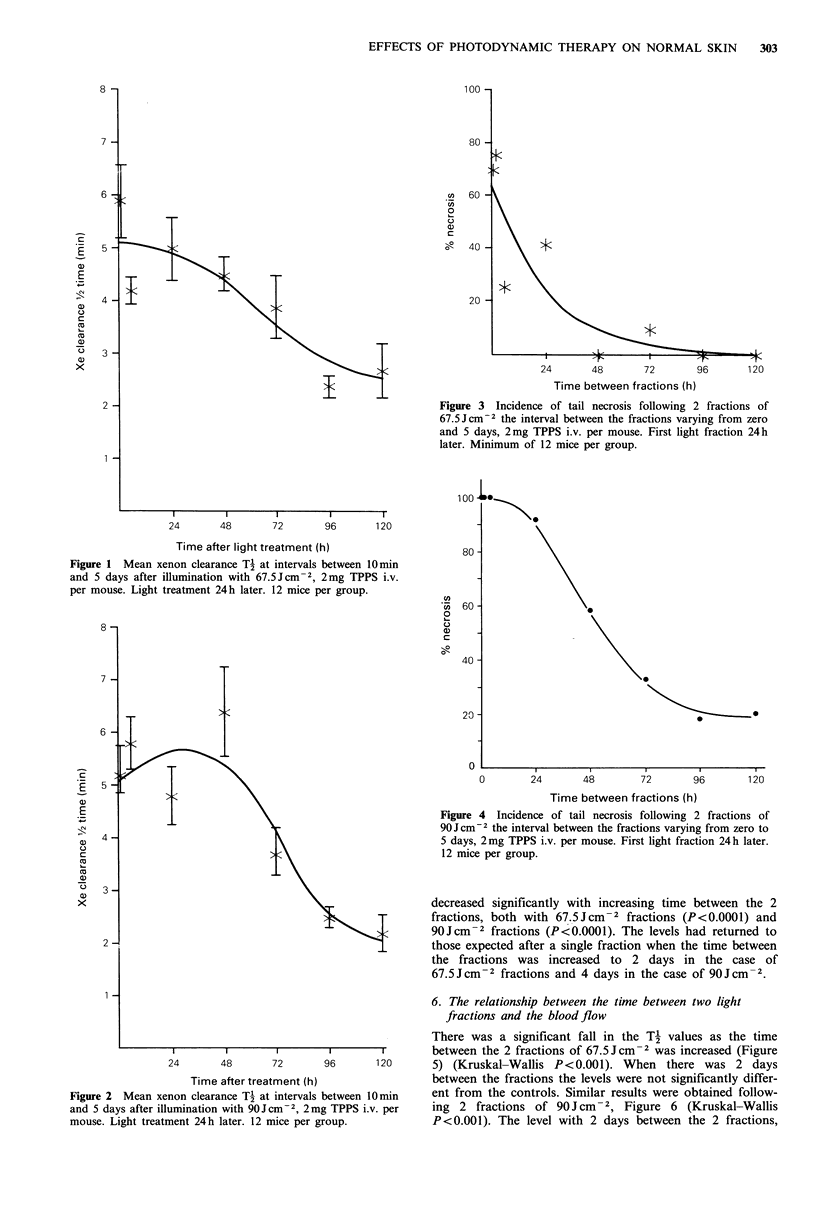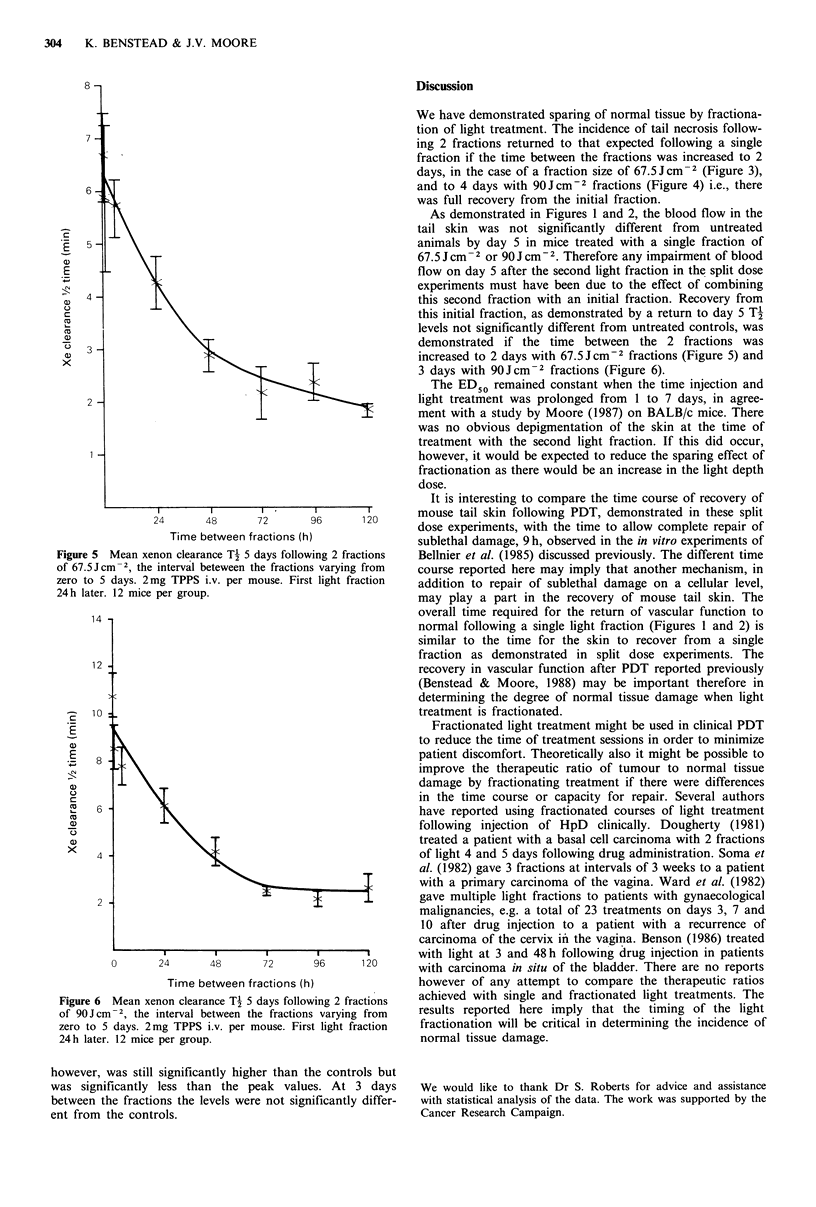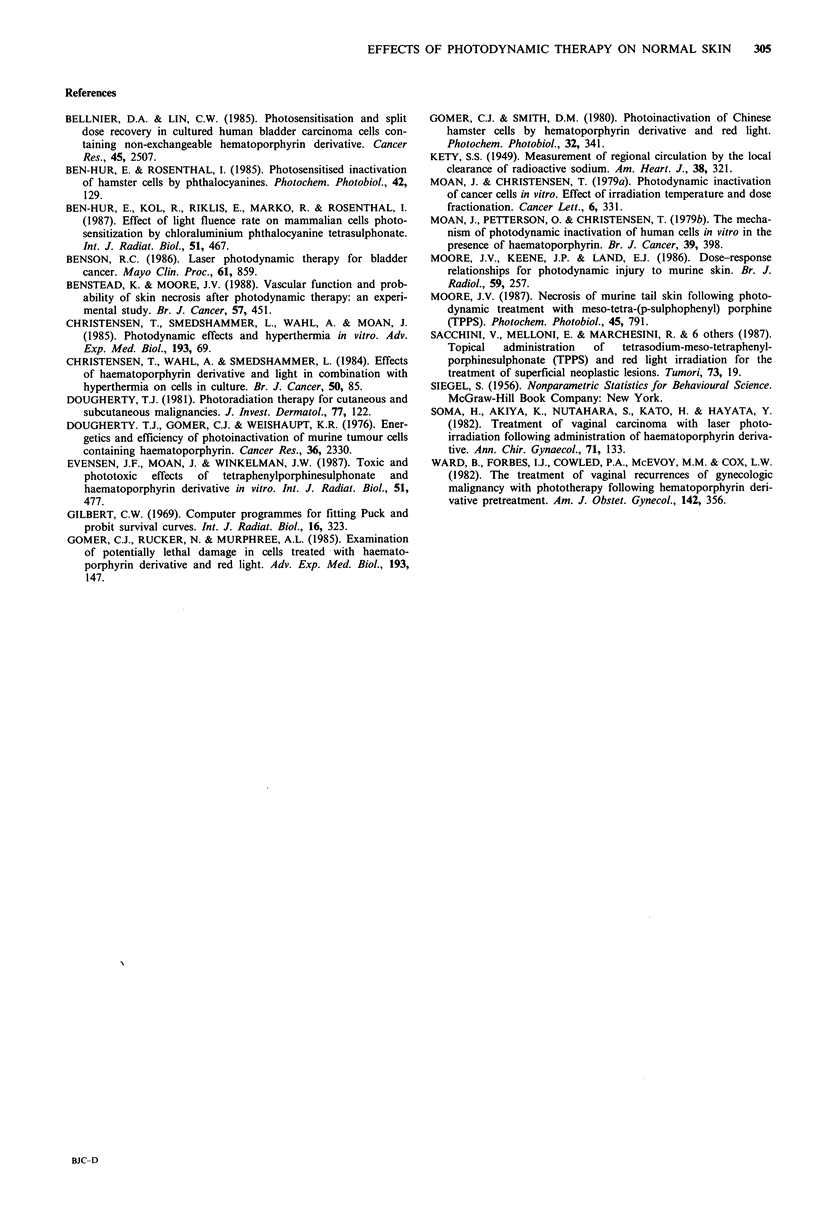# The effect of fractionation of light treatment on necrosis and vascular function of normal skin following photodynamic therapy.

**DOI:** 10.1038/bjc.1988.208

**Published:** 1988-09

**Authors:** K. Benstead, J. V. Moore

**Affiliations:** Paterson Institute for Cancer Research, Christie Hospital and Holt Radium Institute, UK.

## Abstract

Sparing of normal tissue, mouse tail skin, by fractionation of light treatment in photodynamic therapy has been demonstrated in BDF1 mice injected with 2 mg tetrasodium-meso-tetra(4-sulphophenyl)porphine dodecahydrate i.v. When the time between 2 fractions of 67.5 J cm-2 and 90 J cm-2 was increased to 2 and 4 days respectively the incidence of necrosis fell to that expected after a single fraction. Blood flow in the tail skin 5 days after the second light fraction, as measured by the clearance of an intradermally injected solution of 133xenon in 0.9% saline, returned to control values when the time between 2 fractions was 2 days with 67.5 J cm-2 fractions, and 3 days with 90 J cm-2 fractions. The time course of recovery of normal mouse tail skin from photodynamic therapy, as shown by these split dose experiments, was found to be similar to the time course for the recovery of blood flow following a single light treatment.


*Br. J. Cancer* (1988), **58**, 301–305 © The Macmillan Press Ltd., 1988

### The effect of fractionation of light treatment on necrosis and vascular function of normal skin following photodynamic therapy

K. Benstead* & J.V. Moore

*Paterson Institute for Cancer Research, Christie Hospital and Holt Radium Institute, Manchester M20 9BX, UK.*

**Summary** Sparing of normal tissue, mouse tail skin, by fractionation of light treatment in photodynamic therapy has been demonstrated in BDF1 mice injected with 2 mg tetrasodium-meso-tetra(4-sulphophenyl) porphine dodecahydrate i.v. When the time between 2 fractions of 67.5 J cm$^{-2}$ and 90 J cm$^{-2}$ was increased to 2 and 4 days respectively the incidence of necrosis fell to that expected after a single fraction. Blood flow in the tail skin 5 days after the second light fraction, as measured by the clearance of an intradermally injected solution of $^{133}$xenon in 0.9% saline, returned to control values when the time between 2 fractions was 2 days with 67.5 J cm$^{-2}$ fractions, and 3 days with 90 J cm$^{-2}$ fractions. The time course of recovery of normal mouse tail skin from photodynamic therapy, as shown by these split dose experiments, was found to be similar to the time course for the recovery of blood flow following a single light treatment.

Photodynamic therapy (PDT) is based on the selective retention of certain photosensitising drugs in tumours. Exposure of these tumours to light results in activation of the drug and destruction of the tumour cells. There is evidence that mammalian cell lines may accumulate 'sublethal damage' on exposure to PDT. Many workers report an initial shoulder region on curves of cell survival *vs.* light dose, obtained *in vitro*. This has been found in experiments using benign cell lines, e.g., Chinese hamster ovary cells (Gomer & Smith, 1980) and human lymphocytes (Ben Hur *et al.*, 1987) and for cells derived from malignancies, e.g., NHIK cells from carcinoma *in situ* of the cervix (Moan *et al.*, 1979*a*) and mouse mammary carcinoma cells (Dougherty *et al.*, 1976). It has also been reported in experiments using different photosensitising drugs, e.g., haematoporphyrin (HP; Moan *et al.*, 1979*b*), haematoporphyrin derivative (HPD; Christensen *et al.*, 1984), meso-tetra sulphophenyl porphine (TPPS; Evensen *et al.*, 1987) and chloroaluminium phthalocyanine (Ben Hur *et al.*, 1985).

Evidence that environmental conditions may modulate the expression of this sublethal damage initially came from studies on the effect of temperature on the shapes of cell survival curves following PDT. Moan *et al.* (1979*a*) noted that following exposure to HP, irradiation of NHIK cells *in vitro* at 4°C resulted in more efficient inactivation than irradiation at 37°C and there was no shoulder on the cell survival curve. Subsequently, Gomer *et al.* (1985) demonstrated an inhibition of repair of 'potentially lethal damage' when Chinese hamster lung fibroblasts that had received prolonged exposure to HPD, were held post-irradiation at 4°C or in the presence of the metabolic inhibitor caffeine.

Attempts to measure repair of sub-lethal damage by split-dose experiments *in vitro* have been reported by Moan *et al.* (1979*a*), who found that for NHIK cells exposed to light in the presence of HP, a given total light dose was *more* efficient when it was fractionated than when given in a single exposure. Subsequently however, Christensen *et al.* (1985) used the same cell line to demonstrate that relative survival after two doses was a complex function of the interval between doses and that 'sparing' occurred with a 3 h interval. Bellnier & Lin (1985) found that survival of EJ human urinary bladder carcinoma cells rapidly increased with interval between two light fractions, reaching a maximum at 9 h.

The aim of the present study was to determine whether or not fractionation of light treatment resulted in sparing of tissue *in vivo*, using as a model normal mouse tail skin. The photosensitiser employed was TPPS, in which there has been a recent renewal of interest, both experimentally (e.g. Evensen *et al.*, 1987) and clinically (Sacchini *et al.*, 1987). Tissue injury was assessed using two endpoints:

1. the incidence of tail necrosis, as described by Moore *et al.* (1986); and
2. blood flow in the mouse tail skin 5 days after a second light fraction as measured by the clearance of $^{133}$xenon injected intradermally. There is evidence that impairment of vascular function is an important mechanism resulting in damage to both malignant and normal tissues. A previous study found an initial impairment in blood flow following PDT but this returned to normal by day 5 at low light doses. However, at light doses greater than the threshold for skin necrosis, there was increasing impairment of blood flow on day 5 (Benstead & Moore, 1988).

### Materials and methods

### *Mice*

9–12 week old male mice of the pigmented inbred strain B6D2F1 were used. The animals were housed in subdued lighting conditions under a 12 h dark (1800–0600) 12 h light regimen and were supplied with food and water *ad libitum*.

### *Drug*

Tetrasodium-meso-tetra (4-sulphophenyl) porphine dodecahydrate (TPPS; Strem Chemicals, Newburyport, MA), a hydrophilic agent, was dissolved in 0.9% saline. The purity of the compound was >95%, with water and twice-substituted products as impurities (manufacturer's information). A dose of 2 mg in a volume of 0.2 ml was injected as a bolus via the lateral tail vein. This corresponds to a well-tolerated dose of 80 mg kg$^{-1}$, which is less than one third of the $LD_{10}$ dose in these mice (Moore, unpublished). The animals were then housed in the dark for 24 h.

### *Light source*

A 100 W, 12 V quartz tungsten halogen lamp (Xenophot HLX; Wotan, London) was used with a KG1 infra-red filter (Schott, Mainz). This produced a continuous spectrum over the range 300–1100 nm with peak spectral irradiance at approximately 700 nm. Optical lenses produced a circular beam of uniform irradiance over a 2.5 cm diameter (maximum fall off was 10%). The power density on the central axis at the treatment distance was 75 mW cm$^{-2}$.

### *Light treatment*

The animals were lightly restrained without anaesthesia in a

*Present address: Radiotherapy Dept., Addenbrooke's Hospital, Hills Rd., Cambridge CB2 2QQ, UK.
Correspondence: K. Benstead.
Received 22 February 1988; and in revised form, 17 May 1988.

perspex container. The tube containing the tail was covered with black tape apart from the central 2.5 cm. The container was positioned with the central part of the tail across the diameter of the light beam. Surface temperature during illumination was measured with a thermocouple and was not found to rise above 32.5°C.

*Xenon clearance*

The use of the xenon clearance technique for measurement of blood flow in mouse tail skin following PDT has been described previously (Benstead & Moore, 1988). In the experiments reported here blood flow in the tails was stimulated 15 min before and during measurement by raising ambient temperature to 37°C. The mice were restrained in a perspex container and 5 $\mu$l of $^{133}$xenon in 0.9% saline was injected intradermally into the distal end of the treated area. The injection site was positioned under the centre of a scintillation counter attached to a ratemeter and the activity was recorded at 2 minute intervals for a minimum of 10 min. The slope of the line, obtained when the logarithm of the remaining activity was plotted against time, was a function of local blood flow (Kety, 1949). Results were analysed by a computer programme to obtain the least-squares best fit for the exponential half-time ($T\frac{1}{2}$) for xenon clearance.

*Experimental design*

*1. The relationship of the interval between injection and light treatment to the probability of necrosis* There were 6 mice in each experimental group and the experiments were repeated once, the data being pooled. Groups of mice were treated with doses of light in the range 90–202.5 J cm$^{-2}$ either 24 h or 7 days following injection of TPPS. Mice were kept for 30 days and the proportion in which the tail was lost distal to the proximal edge of the light beam was recorded.

*2. The probability of necrosis following a single light treatment* There were 12 mice in each experimental group. Twenty-four hours after drug injection the mice were treated with either 67.5 J cm$^{-2}$ or 90 J cm$^{-2}$. The incidence of tail necrosis was recorded as above.

*3. The time course of impairment and recovery of blood flow following a single light fraction* There were 12 mice in each experimental group. Twenty-four hours after injection of TPPS the mice were treated with either 67.5 J cm$^{-2}$ or 90 J cm$^{-2}$. These doses were chosen as a single fraction was expected to produce a low incidence of necrosis while two consecutive fractions i.e., 135 J cm$^{-2}$ and 180 J cm$^{-2}$, were expected to produce a 50% and 100% incidence of tail necrosis respectively. Values of xenon clearance $T\frac{1}{2}$ were then determined for different groups of animals at 10 min, 4 h, 1, 2, 3, 4 and 5 days following light treatment. A previous study led us to expect that the xenon clearance $T\frac{1}{2}$ would return to control values by day 5 (Benstead & Moore, 1988).

*4. The relationship of the time between 2 light fractions and the probability of necrosis* There were a minimum of 12 mice in each experimental group. Each group was treated with 2 fractions of either 67.5 J cm$^{-2}$ or 90 J cm$^{-2}$. The first fraction was administered 24 h following injection of TPPS. The proportion of tails which necrosed was determined for times between the 2 fractions of 0 min (i.e., a single fraction of 135 J cm$^{-2}$ or 180 J cm$^{-2}$) 10 min, 4 h, 1, 2, 3, 4 and 5 days.

*5. The relationship of the time between 2 light fractions and the blood flow* There were 12 mice in each experimental group. After treatment with 2 light fractions of either 67.5 J cm$^{-2}$ or 90 J cm$^{-2}$, the first fraction being administered 24 h after drug injection, the xenon clearance $T\frac{1}{2}$ was determined for each mouse 5 days after the second light fraction. The time between the 2 fractions was varied from zero to 5 days as in the necrosis end-point experiments.

*6. Controls* Three sets of controls were used. There were a minimum of 24 animals in each control group. (i) Xenon clearance was performed in untreated mice. (ii) Mice were injected with TPPS and the xenon clearance was measured 48 h later. (iii) Mice were treated with 225 J cm$^{-2}$ and the xenon clearance determined 24 h later. All the control animals were observed for 30 days.

*Statistical analysis*

Data comparing incidence of necrosis with light dose were analysed by a probit fitting programme (Gilbert, 1969) to yield values for the ED50, i.e., the light dose that causes a 50% incidence of necrosis in a group of mice.

Values for xenon clearance $T\frac{1}{2}$ were normally distributed in the control groups and were compared by one-way analysis of variance. The results were positively skewed in some of the groups treated with PDT. The data were therefore analysed by the Kruskal–Wallis test which, if significant, was followed by multiple Mann–Whitney U tests using a reduced significance level which allowed us to detect where the differences between the groups were (Siegel, 1956).

Values for the number of tails undergoing necrosis with different times between light fractions were compared using the Chi-square test.

### Results

*1. Controls*

There was no necrosis in the control groups. The mean value for the xenon clearance $T\frac{1}{2}$ at 24 h for animals treated with light only (2.9 $\pm$ 0.92 min; error as 1 s.d.) and at 48 h for animals injected with 2 mg TPPS only (2.4 $\pm$ 0.77 min) were not significantly different from the control group which had received no treatment (2.6 $\pm$ 0.57 min). Groups treated with both drug and light were compared statistically with untreated controls.

*2. The relationship of the time between injection and light administration to the probability of necrosis*

Probit analysis of the incidence of necrosis *vs.* the light dose yielded an ED50 value of 137 $\pm$ 10 J cm$^{-2}$ (error as 1 s.e.) for tails exposed to light 24 h after drug injection. Light treatment 7 days after drug administration yielded an ED50 value of 137 $\pm$ 6 J cm$^{-2}$. There was no significant difference between these 2 curves.

*3. The probability of necrosis following a single light treatment*

Tail necrosis occurred in 1 of the 12 mice treated with 67.5 J cm$^{-2}$ and 2 of the 12 mice treated with 90 J cm$^{-2}$.

*4. The time course of impairment and recovery of blood flow following a single light fraction*

Figures 1 and 2 show the mean xenon clearance $T\frac{1}{2}$ for different times between 10 min and 5 days following light doses of 67.5 and 90 J cm$^{-2}$ respectively. The rise in the $T\frac{1}{2}$ at 10 min following PDT with 67.5 J cm$^{-2}$ was significant (Kruskal–Wallis $P<0.01$). The levels then fell and by day 3 were not significantly different than the control group. The pattern observed following PDT with a light dose of 90 J cm$^{-2}$ was similar (Kruskal–Wallis $P<0.01$) with a significant rise in the $T\frac{1}{2}$ values at 10 min. The values peaked at day 2 and there was a significant fall on day 3 although at this time the values were still significantly higher than group control. By days 4 and 5 the levels were not significantly different than the controls.

*5. The relationship of the time between 2 light fractions and the probability of necrosis*

As shown in Figures 3 and 4, the incidence of tail necrosis

**Figure 1** Mean xenon clearance T½ at intervals between 10 min and 5 days after illumination with 67.5 J cm$^{-2}$, 2 mg TPPS i.v. per mouse. Light treatment 24 h later. 12 mice per group.

**Figure 2** Mean xenon clearance T½ at intervals between 10 min and 5 days after illumination with 90 J cm$^{-2}$, 2 mg TPPS i.v. per mouse. Light treatment 24 h later. 12 mice per group.

**Figure 3** Incidence of tail necrosis following 2 fractions of 67.5 J cm$^{-2}$ the interval between the fractions varying from zero and 5 days, 2 mg TPPS i.v. per mouse. First light fraction 24 h later. Minimum of 12 mice per group.

**Figure 4** Incidence of tail necrosis following 2 fractions of 90 J cm$^{-2}$ the interval between the fractions varying from zero to 5 days, 2 mg TPPS i.v. per mouse. First light fraction 24 h later. 12 mice per group.

decreased significantly with increasing time between the 2 fractions, both with 67.5 J cm$^{-2}$ fractions ($P<0.0001$) and 90 J cm$^{-2}$ fractions ($P<0.0001$). The levels had returned to those expected after a single fraction when the time between the fractions was increased to 2 days in the case of 67.5 J cm$^{-2}$ fractions and 4 days in the case of 90 J cm$^{-2}$.

*6. The relationship between the time between two light fractions and the blood flow*

There was a significant fall in the T½ values as the time between the 2 fractions of 67.5 J cm$^{-2}$ was increased (Figure 5) (Kruskal–Wallis $P<0.001$). When there was 2 days between the fractions the levels were not significantly different from the controls. Similar results were obtained following 2 fractions of 90 J cm$^{-2}$, Figure 6 (Kruskal–Wallis $P<0.001$). The level with 2 days between the 2 fractions,

**Figure 5** Mean xenon clearance T½ 5 days following 2 fractions of 67.5 J cm$^{-2}$, the interval beteween the fractions varying from zero to 5 days. 2 mg TPPS i.v. per mouse. First light fraction 24 h later. 12 mice per group.

**Figure 6** Mean xenon clearance T½ 5 days following 2 fractions of 90 J cm$^{-2}$, the interval between the fractions varying from zero to 5 days. 2 mg TPPS i.v. per mouse. First light fraction 24 h later. 12 mice per group.

however, was still significantly higher than the controls but was significantly less than the peak values. At 3 days between the fractions the levels were not significantly different from the controls.

### Discussion

We have demonstrated sparing of normal tissue by fractionation of light treatment. The incidence of tail necrosis following 2 fractions returned to that expected following a single fraction if the time between the fractions was increased to 2 days, in the case of a fraction size of 67.5 J cm$^{-2}$ (Figure 3), and to 4 days with 90 J cm$^{-2}$ fractions (Figure 4) i.e., there was full recovery from the initial fraction.

As demonstrated in Figures 1 and 2, the blood flow in the tail skin was not significantly different from untreated animals by day 5 in mice treated with a single fraction of 67.5 J cm$^{-2}$ or 90 J cm$^{-2}$. Therefore any impairment of blood flow on day 5 after the second light fraction in the split dose experiments must have been due to the effect of combining this second fraction with an initial fraction. Recovery from this initial fraction, as demonstrated by a return to day 5 T½ levels not significantly different from untreated controls, was demonstrated if the time between the 2 fractions was increased to 2 days with 67.5 J cm$^{-2}$ fractions (Figure 5) and 3 days with 90 J cm$^{-2}$ fractions (Figure 6).

The $ED_{50}$ remained constant when the time injection and light treatment was prolonged from 1 to 7 days, in agreement with a study by Moore (1987) on BALB/c mice. There was no obvious depigmentation of the skin at the time of treatment with the second light fraction. If this did occur, however, it would be expected to reduce the sparing effect of fractionation as there would be an increase in the light depth dose.

It is interesting to compare the time course of recovery of mouse tail skin following PDT, demonstrated in these split dose experiments, with the time to allow complete repair of sublethal damage, 9 h, observed in the *in vitro* experiments of Bellnier *et al.* (1985) discussed previously. The different time course reported here may imply that another mechanism, in addition to repair of sublethal damage on a cellular level, may play a part in the recovery of mouse tail skin. The overall time required for the return of vascular function to normal following a single light fraction (Figures 1 and 2) is similar to the time for the skin to recover from a single fraction as demonstrated in split dose experiments. The recovery in vascular function after PDT reported previously (Benstead & Moore, 1988) may be important therefore in determining the degree of normal tissue damage when light treatment is fractionated.

Fractionated light treatment might be used in clinical PDT to reduce the time of treatment sessions in order to minimize patient discomfort. Theoretically also it might be possible to improve the therapeutic ratio of tumour to normal tissue damage by fractionating treatment if there were differences in the time course or capacity for repair. Several authors have reported using fractionated courses of light treatment following injection of HpD clinically. Dougherty (1981) treated a patient with a basal cell carcinoma with 2 fractions of light 4 and 5 days following drug administration. Soma *et al.* (1982) gave 3 fractions at intervals of 3 weeks to a patient with a primary carcinoma of the vagina. Ward *et al.* (1982) gave multiple light fractions to patients with gynaecological malignancies, e.g. a total of 23 treatments on days 3, 7 and 10 after drug injection to a patient with a recurrence of carcinoma of the cervix in the vagina. Benson (1986) treated with light at 3 and 48 h following drug injection in patients with carcinoma *in situ* of the bladder. There are no reports however of any attempt to compare the therapeutic ratios achieved with single and fractionated light treatments. The results reported here imply that the timing of the light fractionation will be critical in determining the incidence of normal tissue damage.

We would like to thank Dr S. Roberts for advice and assistance with statistical analysis of the data. The work was supported by the Cancer Research Campaign.

### References

BELLNIER, D.A. & LIN, C.W. (1985). Photosensitisation and split dose recovery in cultured human bladder carcinoma cells containing non-exchangeable hematoporphyrin derivative. *Cancer Res.*, **45**, 2507.

BEN-HUR, E. & ROSENTHAL, I. (1985). Photosensitised inactivation of hamster cells by phthalocyanines. *Photochem. Photobiol.*, **42**, 129.

BEN-HUR, E., KOL, R., RIKLIS, E., MARKO, R. & ROSENTHAL, I. (1987). Effect of light fluence rate on mammalian cells photosensitization by chloraluminium phthalocyanine tetrasulphonate. *Int. J. Radiat. Biol.*, **51**, 467.

BENSON, R.C. (1986). Laser photodynamic therapy for bladder cancer. *Mayo Clin. Proc.*, **61**, 859.

BENSTEAD, K. & MOORE, J.V. (1988). Vascular function and probability of skin necrosis after photodynamic therapy: an experimental study. *Br. J. Cancer*, **57**, 451.

CHRISTENSEN, T., SMEDSHAMMER, L., WAHL, A. & MOAN, J. (1985). Photodynamic effects and hyperthermia *in vitro*. *Adv. Exp. Med. Biol.*, **193**, 69.

CHRISTENSEN, T., WAHL, A. & SMEDSHAMMER, L. (1984). Effects of haematoporphyrin derivative and light in combination with hyperthermia on cells in culture. *Br. J. Cancer*, **50**, 85.

DOUGHERTY, T.J. (1981). Photoradiation therapy for cutaneous and subcutaneous malignancies. *J. Invest. Dermatol.*, **77**, 122.

DOUGHERTY. T.J., GOMER, C.J. & WEISHAUPT, K.R. (1976). Energetics and efficiency of photoinactivation of murine tumour cells containing haematoporphyrin. *Cancer Res.*, **36**, 2330.

EVENSEN, J.F., MOAN, J. & WINKELMAN, J.W. (1987). Toxic and phototoxic effects of tetraphenylporphinesulphonate and haematoporphyrin derivative *in vitro*. *Int. J. Radiat. Biol.*, **51**, 477.

GILBERT, C.W. (1969). Computer programmes for fitting Puck and probit survival curves. *Int. J. Radiat. Biol.*, **16**, 323.

GOMER, C.J., RUCKER, N. & MURPHREE, A.L. (1985). Examination of potentially lethal damage in cells treated with haematoporphyrin derivative and red light. *Adv. Exp. Med. Biol.*, **193**, 147.

GOMER, C.J. & SMITH, D.M. (1980). Photoinactivation of Chinese hamster cells by hematoporphyrin derivative and red light. *Photochem. Photobiol.*, **32**, 341.

KETY, S.S. (1949). Measurement of regional circulation by the local clearance of radioactive sodium. *Am. Heart. J.*, **38**, 321.

MOAN, J. & CHRISTENSEN, T. (1979*a*). Photodynamic inactivation of cancer cells *in vitro*. Effect of irradiation temperature and dose fractionation. *Cancer Lett.*, **6**, 331.

MOAN, J., PETTERSON, O. & CHRISTENSEN, T. (1979*b*). The mechanism of photodynamic inactivation of human cells *in vitro* in the presence of haematoporphyrin. *Br. J. Cancer*, **39**, 398.

MOORE, J.V., KEENE, J.P. & LAND, E.J. (1986). Dose–response relationships for photodynamic injury to murine skin. *Br. J. Radiol.*, **59**, 257.

MOORE, J.V. (1987). Necrosis of murine tail skin following photodynamic treatment with meso-tetra-(p-sulphophenyl) porphine (TPPS). *Photochem. Photobiol.*, **45**, 791.

SACCHINI, V., MELLONI, E. & MARCHESINI, R. & 6 others (1987). Topical administration of tetrasodium-meso-tetraphenylporphinesulphonate (TPPS) and red light irradiation for the treatment of superficial neoplastic lesions. *Tumori*, **73**, 19.

SIEGEL, S. (1956). *Nonparametric Statistics for Behavioural Science*. McGraw-Hill Book Company: New York.

SOMA, H., AKIYA, K., NUTAHARA, S., KATO, H. & HAYATA, Y. (1982). Treatment of vaginal carcinoma with laser photoirradiation following administration of haematoporphyrin derivative. *Ann. Chir. Gynaecol.*, **71**, 133.

WARD, B., FORBES, I.J., COWLED, P.A., McEVOY, M.M. & COX, L.W. (1982). The treatment of vaginal recurrences of gynecologic malignancy with phototherapy following hematoporphyrin derivative pretreatment. *Am. J. Obstet. Gynecol.*, **142**, 356.